# Lipid profiling in maternal and fetal circulations in preeclampsia and fetal growth restriction-a prospective case control observational study

**DOI:** 10.1186/s12884-020-2753-1

**Published:** 2020-01-30

**Authors:** Thushari I. Alahakoon, Heather J. Medbury, Helen Williams, Vincent W. Lee

**Affiliations:** 1 University of Sydney, Sydney Medical School, Sydney, NSW Australia; 2 Westmead Institute for Maternal and Fetal Medicine, Westmead Hospital, Westmead, NSW Australia; 3 Department of Surgery, Westmead Hospital, Westmead, NSW Australia; 4 Department of Renal Medicine, Westmead Hospital and University of Sydney, Westmead, NSW Australia

**Keywords:** Preeclampsia, Fetal growth restriction, Maternal lipids, Fetal lipids, Apolipoprotein

## Abstract

**Background:**

While many risk factors for preeclampsia, such as increased body mass index, advanced maternal age, chronic hypertension, diabetes, are now established in clinical practice, maternal lipid profile has not been included in the risk assessment for preeclampsia. We aim to characterize the serum levels of Total Cholesterol (TC), High density lipoprotein (HDL), Low density lipoprotein (LDL), Triglycerides (TG), Apolipoprotein A1, Apolipoprotein B and their ratios TC/HDL and ApoB/ApoA1 in the maternal and fetal circulations of normal pregnancy, preeclampsia (PE), fetal growth restriction (FGR) and PE + FGR.

**Methods:**

A prospective cross-sectional case control study was conducted measuring maternal and fetal lipid levels by enzymatic analysis and immune-turbidimetric enzymatic assays. FGR was defined by elevated umbilical artery Doppler resistance in association with estimated fetal weight < 10%. Kruskal Wallis non-parametric analysis of variance was used to test for homogeneity across the clinical groups for each of the variables, Mann-Whitney tests for pairwise comparisons and Spearman rank correlation were used to quantify gestational age-related changes.

**Results:**

(1) TG levels were elevated in maternal PE and cord blood PE + FGR groups compared to normal pregnancies. (2) A statistically significant elevation of fetal ApoB levels was observed in PE, FGR and PE + FGR compared to normal pregnancies. Apolipoprotein levels A1 and B were not different between maternal groups. (3) TC, HDL, LDL and TC/HDL levels did not show any significant gestational variation or between clinical groups in the maternal or fetal circulation.

**Conclusions:**

Elevation in maternal TG levels may have a role in the pathogenesis of PE. The implications of elevated maternal and fetal TG levels and elevated fetal Apolipoprotein B levels deserves further exploration of their role in long term cardiovascular risk in the mother as well as the offspring.

## Background

Preeclampsia (PE) and fetal growth restriction (FGR) have maternal and fetal implications for pregnancy as well for the long-term health. The elevated risk of hypertension, ischaemic heart disease, stroke, venous thromboembolism [1, 2], renal disease [3] and vascular dementia [4] in women who develop PE is well documented. The long term increased cardiovascular disease (CVD) risk in persons with a history of fetal and neonatal growth restriction is also well established [5]. While many risk factors for preeclampsia, such as increased body mass index, advanced maternal age, chronic hypertension, diabetes, are now established in clinical practice [6], maternal lipid profile has not been included in the risk assessment for preeclampsia such as the Fetal Medicine Foundation algorithm [7]. A combination of maternal, historical, ultrasound and biomarkers are currently used clinically in screening for preeclampsia [8].

Hypercholesterolemia and hyperlipidemia are strongly associated with CVD as they promote atherosclerosis, a precursor to myocardial infarction, stroke, and peripheral vascular disease [9]. Lipid profile including total cholesterol (TC), high density cholesterol (HDL) and triglycerides (TG) serves as a screening tool for dyslipidemia and the risk of CVD. Using these values low density lipoprotein (LDL) and total cholesterol/ HDL ratio (TC/HDL) are calculated. HDL and its major protein ApolipoproteinA1 (ApoA1) are recognized as independent protective factors against coronary heart disease [10], while elevated Apolipoprotein B (ApoB), LDL and TG are associated with a higher risk of atherosis and cardiovascular disease [11–14].

Triglycerides are a commonly measured component of lipid profiles for cardiovascular risk assessment [15]. Raised triglycerides are strongly associated with future risk of diabetes as well as cardiovascular disease [16, 17] with elevated TG suggested as an explanation for residual cardiovascular risk even after statin therapy [18]. ApoB and the ApoB/ApoA1 ratios have been advocated as better markers of risk of vascular disease, therapeutic targets in managing patients on lipid lowering therapy [19] and a better guide to the adequacy of statin treatment than any cholesterol index [20–22].

All lipids and apolipoproteins have been shown to be significantly elevated in pregnancy, the most prominent change being a 2.7-fold increase in triglycerides in the third trimester [23–27]. As pregnancy progresses, lipids levels steadily increase during the pregnancy with a noticeable increase in the third trimester [28]. This lipid metabolism throughout pregnancy allows for proper nutrients for the fetus. Studies have demonstrated antenatal serum TG and free fatty acid concentrations to be increased approximately twofold in women with preeclampsia relative to uncomplicated pregnancies with no effect on total cholesterol, HDL, and LDL [29]. The atherogenic indices TC/HDL, ApoB/ApoA1 and LDL/HDL have been shown to be reduced in first trimester and elevated in third trimester - however none of the lipoprotein components have been correlated with the age and parity of the pregnant women [23].

ApoA1 and ApoB levels have also been found not to be different in normal versus preeclamptic pregnancies [30]. Early pregnancy dyslipidemia including increased serum TG levels in early pregnancy before 20 weeks and elevated oxidized-LDL are associated with an increased risk of preeclampsia [31–34].

A role for the lipid status and metabolic milieu of the mother has been proposed as a contributor for the clinically diverse presentations of PE and FGR in the mother with similar placental disease as well as for the long-term cardiovascular risks in preeclampsia [35].

Published studies have been on small for gestational age (SGA) fetuses at estimated birth weight less than 10th centile as well as FGR defined by estimated fetal weight less than 10th centile with abnormal umbilical or uterine Doppler studies or estimated fetal weight less than 3rd centile [36]. A study on maternal cholesterol in PE and FGR found LDL and TC concentrations to be significantly lower in the FGR compared to the normal pregnancy [37].

During late gestation, enhanced lipolytic activity in adipose tissue in the mother contributes to maternal hyperlipidaemia [38]. There is an increase in plasma triacylglycerol, phospholipid and cholesterol concentrations. Lipoprotein receptors in the placenta allows their placental uptake, where they are hydrolysed by lipoprotein lipase, phospholipase A [2] and intracellular lipase and the released fatty acids available for fetal metabolism. Maternal plasma non-esterified fatty acids are transferred across the placenta and provide a source of long chain polyunsaturated fatty acids for the fetus. Placental transfer of glycerol is not documented. Although maternal cholesterol contributes to fetal cholesterol during early pregnancy, fetal cholesterol biosynthesis rather than cholesterol transfer from maternal lipoproteins seems to be the main mechanism for satisfying fetal requirements during late pregnancy [38].

Lipid profile of the fetal and placental circulations in normal pregnancy have been mentioned as part of investigation into FGR [37, 39] but have not been well characterized. A comparison of circulating ApoA1 and ApoB concentrations in fetal umbilical cord plasma samples obtained at diagnostic cordocentesis of growth-retarded compared with normal fetuses found that while there were no differences in median plasma ApoA1 concentrations between the groups, plasma ApoB levels and the ApoB/A1 ratio were significantly higher in growth-retarded fetuses [40]. A recent study described elevated ApoB content in very low density lipoprotein particles and a reduction of ApoA1 content in HDL particles in SGA neonates [41]. Apolipoproteins in cord blood of preeclamptic pregnancies have not been described.

While some investigation into maternal and fetal lipids in the context of PE and FGR has been conducted, none of these studies has run a full lipid panel across different conditions in both maternal and fetal samples. In this study we aimed to examine the serum levels of TC, HDL, LDL, ApoA1, ApoB and their ratios TC/HDL and ApoB/ApoA1 in the maternal and fetal circulations of normal pregnancy, PE, FGR and PE + FGR to assess differences between groups as an insight into the pathogenesis and potential impact on long term outcomes.

## Methods

A prospective cross-sectional case control study was conducted. Pregnant women between 24 and 40 weeks of gestation, as defined by first trimester ultrasound, delivering at an Australian tertiary center during 2013–2014, were recruited from ultrasound service, antenatal ward, and antenatal clinics and classified into four clinical groups of normal pregnancy, PE, FGR and PE + FGR. Written consent was obtained from all participants in the study. The normal pregnancy controls were gestationally matched to the study groups with at least two normal pregnancy samples recruited for each of the pathological pregnancies two-week gestational age ranges.

All patients classified as PE in this study satisfied the ISSHP 2014 criteria for preeclampsia [42]. FGR was defined as birth weight less than 10th centile with elevated umbilical artery Doppler systolic / diastolic ratio or Resistance Index >95th centile for gestation [36]. All patients with PE, PE + FGR or FGR underwent antenatal ultrasound examination after 24 weeks of gestation and within 7 days of delivery using General Electric (GE) Voluson E8 ultrasound equipment. Patients with pre-existing hypertension, renal disease, pre-existing diabetes, gestational diabetes and multiple pregnancies were excluded from the study.

The maternal peripheral venous blood was collected antenatally in third trimester prior to labour or delivery. Fetal cord blood from the umbilical vein was collected at the time of delivery. The lipid profile including TG, TC, HDL, LDL, ApoA1 and ApoB of maternal and fetal samples were tested and the ratios TC/HDL, ApoB/ApoA1 were calculated. Serum was analyzed for cholesterol and triglyceride using enzymatic analysis (Siemens Dimensions Vista system). ApoA1 and ApoB were assayed by immunoturbidimetry (Abbott Diagnostic Architect C4000 Ci 4100). A summary of the techniques, commercial kits and reference values for pregnant and non-pregnant populations for the assays used are listed in Table 1. The estimation of LDL cholesterol was performed in the clinical laboratories using Friedewald equation [43]. Specimens with triglyceride > 4.5 mmol/L were also used in the analysis as maternal triglycerides in normal pregnancy can be over this threshold [23]. The fasting time for the sample collection ranged from 6 h to 12 h. While the TG assays are typically performed after a 10 h fast, there is evidence non-fasting and postprandial triglycerides > 2.5 mmol/L can be considered parallel to the fasting 2.0 mmol/L in assessing the risk of cardiovascular disease [44].

**Table 1:** List of markers in the lipid profile and the commercial assays used for testing the samples. The reference ranges for serum and plasma lipids for non pregnant population as listed in the commercial assays and the 5th, 50th and 95th centile values in third trimester of pregnancy as published by Piechota et al. [23]

Variable	Reference range	Method/Assay	5th, 50th and 95th centile values for the pregnant population
Total Cholesterol (TC) mmol/L	3.0–5.5	Siemens Dimensions Vista system CHOL Automated enzymatic assays K1027	1.35, 7.38, 9.83
High density cholesterol (HDL) mmol/L	≥1.0	Siemens Dimensions Vista system Automated enzymatic assays K3048A	1.04, 1.63, 2.46
Low density cholesterol (LDL) mmol/L	≤3.5	Calculated from TC, HDL and TG TC-HDL- TG/2.2	2.56, 4.31, 6.48
Triglycerides (TG) mmol/L	≤2.0	Siemens Dimensions Vista system Automated enzymatic assays	1.4, 2.63, 4.68
Apolipoprotein A1 (ApoA1) g/L	1.10–1.89	Abbott Diagnostic Architect C4000 Automated immuno-turbidimetric assay	1.42, 2.0, 2.61
Apo lipoprotein B (ApoB) g/L	0.59–1.32	Abbott Diagnostic Architect C4000 Automated immuno-turbidimetric assay	0.89, 1.32, 1.92

### Statistical analysis

The statistical software packages SPSS for windows Version 21 and SPLUS version 8 were used to for data analysis. Two-tailed tests with a 5% significance level were used throughout. Kruskal Wallis non-parametric analysis of variance was used to test for homogeneity across the four clinical groups for each of the variables, TC, HDL, LDL, TC/HDL, ApoA1, ApoB and ApoB/ApoA1 ratio. Where heterogeneity was demonstrated, Mann-Whitney tests were used for pairwise comparisons between normal pregnancy and each of the clinical groups as well as between each of the pathological groups. The Spearman rank correlation was used to quantify the extent of the pairwise association between each of the variables TC, HDL, LDL, TC/HDL, ApoA1, ApoB and ApoB/ApoA1 ratio and gestational age. Box plots were used to illustrate the distribution of continuous variables by patient group. Data are summarized as median and interquartile range. Results are presented as median, upper and lower quartiles. This study was conducted as a pilot study for hypothesis generation and to establish maternal and fetal lipid levels, required to design power calculations for further studies. Due to the small sample numbers in a pilot study, confounders were not statistically adjusted.

## Results

### Maternal and neonatal characteristics of the study population

Maternal fasting venous blood samples were collected from 52 women prior to delivery including Normal pregnancy (*n* = 20), PE (*n* = 10), FGR (*n* = 12) and PE + FGR (*n* = 10). Umbilical vein cord blood sampled from 30 pregnancies was available for analysis (Table 2). The number of fetal samples was limited by premature gestation, small placenta and blood volume, emergency deliveries, inadequate volume available after delayed cord clamping. All women had an unrestricted diet and were fasting for at least 6 h prior to the test. The normal pregnancy patients were gestationally matched to the pathological pregnancies complicated by PE and FGR. The maternal and fetal demographic data for all maternal samples and all fetal samples are presented in Table 2.

**Table 2:** Maternal and fetal demographic data and clinical characteristics of the study population. Results are presented as mean ± SD for each continuous variable unless otherwise specified

	Group			
	Normal	PE only	FGR only	PE + FGR
	Mean (Standard Deviation)	Mean (Standard Deviation)	Mean (Standard Deviation)	Mean (Standard Deviation)
Number of maternal samples	20	10	12	10
Number of cord blood / fetal samples	7	5	8	10
Maternal age (years)	28.8 ± 4.0	27.5 ± 5.8	28.3 ± 4.9	33.2 ± 6.7*^#^†
BMI	27.6 ± 5.4	30.3 ± 8.0	24.1 ± 5.3^#^	28.0 ± 6.0
Gestation age at maternal sample collection (weeks)	36.1 ± 3.8	35.2 ± 3.4	34.8 ± 4.2	31.7 ± 2.8*^#^ †
Gestation age at delivery (weeks) all maternal samples	39.3 ± 0.8	36.5 ± 3.4*	36.2 ± 2.2*	32.0 ± 3.0*^#^ †
Gestation age at delivery (weeks) all fetal samples	39.08 ± 0.44	36.9 ± 2.1	36 ± 2.59	32.0 ± 3.0*^#^ †
Birth weight (g) all maternal samples	3412 ± 388	2897 ± 903*	1831 ± 781*	1398 ± 619*^#^ †
Birth weight (g) all fetal samples	3359 ± 298	3046 ± 533	1981 ± 642	1398 ± 619*^#^ †
Primipara (%)	40	80	58.3	90
Smoking (%)	5.6	0.0	8.3	10
Mode of delivery (LSCS) (%)	75	80	75	80

*Statistically significant difference from normal pregnancy. # statistically significant difference from PE. †Statistically significant difference from FGR

Analysis of the effect of confounding variables using Fisher’s exact test showed that PE + FGR patients were older, were delivered earlier and had lower birth weights. There was no significant difference in BMI between groups except the pregnancies complicated by FGR had a lower BMI as compared to the PE group. No difference was noted in the incidence of smoking and mode of delivery between the different clinical groups. The majority of PE patients received anti-hypertensive treatment whereas only half of the PE + FGR patients were medicated. The gestational age at sample collection in the PE + FGR group was significantly higher than the other three groups of normal, PE and FGR. Significant differences in both gestation at delivery and birth weight seen between normal pregnancy and PE, FGR and PE + FGR, as well as PE + FGR and the other three groups. No difference in gestational age was seen between PE and FGR (Table 2).

Spearman rank correlation of gestational age and the measured variables total cholesterol, HDL, LDL, triglycerides ApoA1 and ApoB for all the study patients including PE, FGR and PE + FGR as well as for normal group only did not show any significant correlation with gestational range 26–40 weeks.

### Maternal lipid profiles in normal and complicated pregnancies

**Fig. 1:**
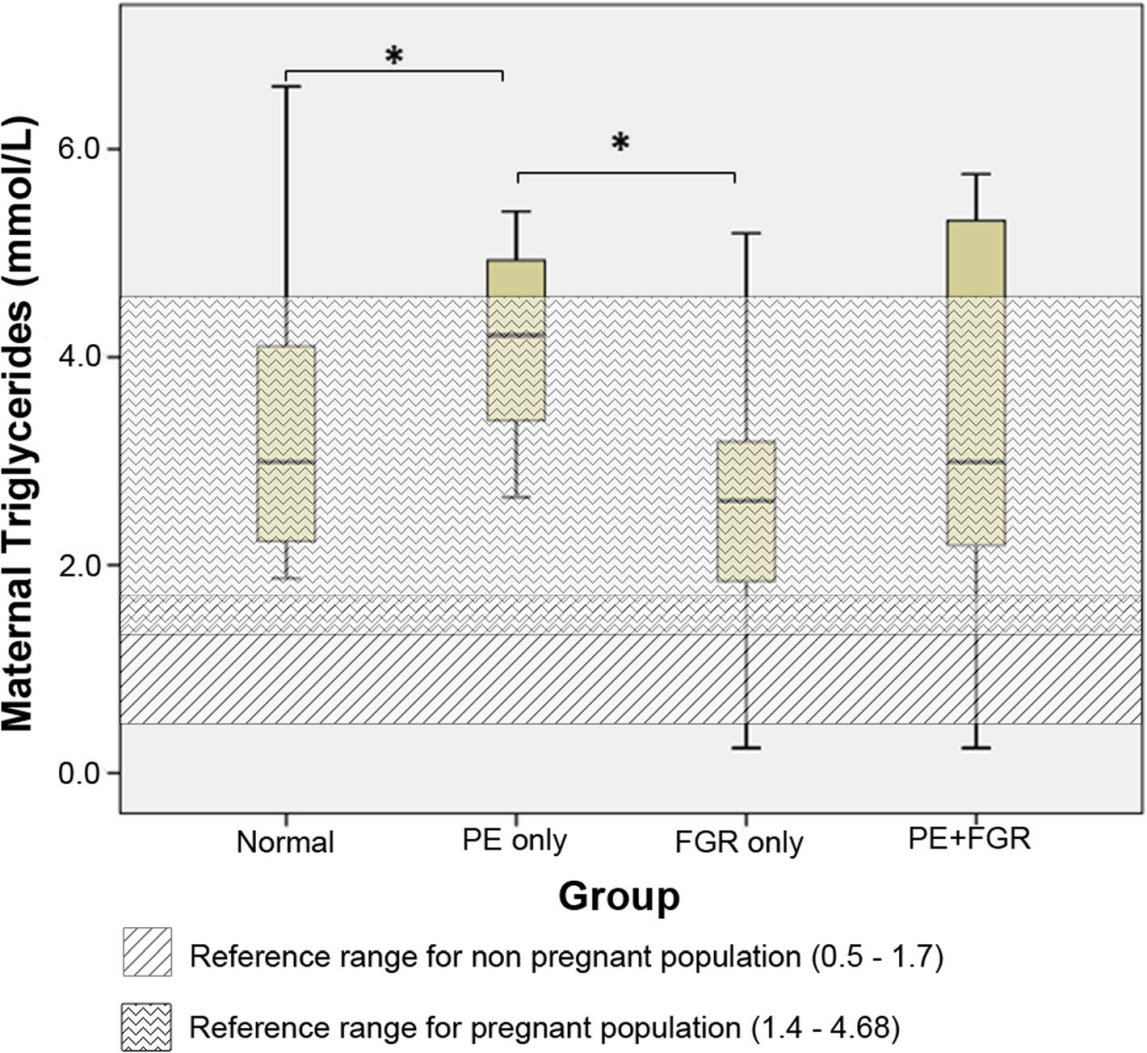
Comparison of maternal total triglyceride (TG) levels between clinical groups. The clinically applied reference range for non-pregnant population and the published TG reference range (5th–95th centile) for normal pregnancy [23] are indicated by the shaded areas

The maternal lipid profiles of each clinical group are summarized in Table 3 and Fig. 1. The clinically applied TG reference range for non-pregnant population (5-95th centile) and the published TG reference range (5th–95th centile) for normal pregnancy [23] are indicated by the shaded area in Fig. 1. The results demonstrate that the TG levels are higher in pregnancy compared to the non-pregnant population normal range. There was a significant increase in the TG levels (*p* < 0.05) in preeclampsia as compared to normal pregnancy and FGR (Fig. 1). No significant differences were noted between the clinical groups for TC, HDL, LDL and Apolipoproteins with the majority of ApoA1 and B readings noted to be lower than the 95th centile (*P* < 0.05) for third trimester pregnancy (Table 3).

**Table 3:** Maternal lipid profile in normal pregnancy and pregnancies complicated by preeclampsia, intrauterine fetal growth restriction and a combination of PE and FGR. Results are presented as median ± interquartile range for each continuous variable

Maternal Data	Group			
	Normal	PE only	FGR only	PE + FGR
	Median (Quartiles 25, 75)	Median (Quartiles 25, 75)	Median (Quartiles 25, 75)	Median (Quartiles 25, 75)
Number of samples	20	10	12	10
TC mmol/L	6.6 (6.1, 7.5)	7.2 (5.9, 8.0)	6.9 (6.0, 7.5)	6.2 (5.5, 6.7)
HDL mmol/L	1.8 (1.6, 1.9)	1.8 (1.5, 2.1)	1.6 (1.3, 1.9)	1.5 (1.3, 2.1)
LDL mmol/L	3.6 (2.4, 4.6)	3.7 (2.2, 4.3)	3.6 (3.0, 4.1)	2.9 (2.6, 3.0)
TC/HDL ratio	3.8 (3.4, 4.7)	3.8 (3.3, 4.9)	4.0 (3.0, 4.7)	3.7 (2.9, 4.9)
TG mmol/L	2.99 (2.23, 4.11)	4.21*† (3.39, 4.93)	2.62 (1.85, 3.19)	2.99 (2.19, 5.31)
Apo A1 g/L	1.91 (1.65, 2.19)	2.06 (1.87, 2.39)	1.87 (1.71, 2.02)	1.91 (1.78, 2.22)
Apo B g/L	1.44 (1.12, 1.82)	1.38 (1.19, 1.63)	1.44 (1.26, 1.53)	1.24 (1.08, 1.53)
Apo B: Apo A1	0.69 (0.60, 0.98)	0.63 (0.59, 0.81)	0.75 (0.59, 0.87)	0.67 (0.47, 0.72)

*Statistically significant difference from normal pregnancy *p* < 0.05. † Statistically significant difference from FGR *p* < 0.05

### Fetal lipid profile

**Fig. 2:**
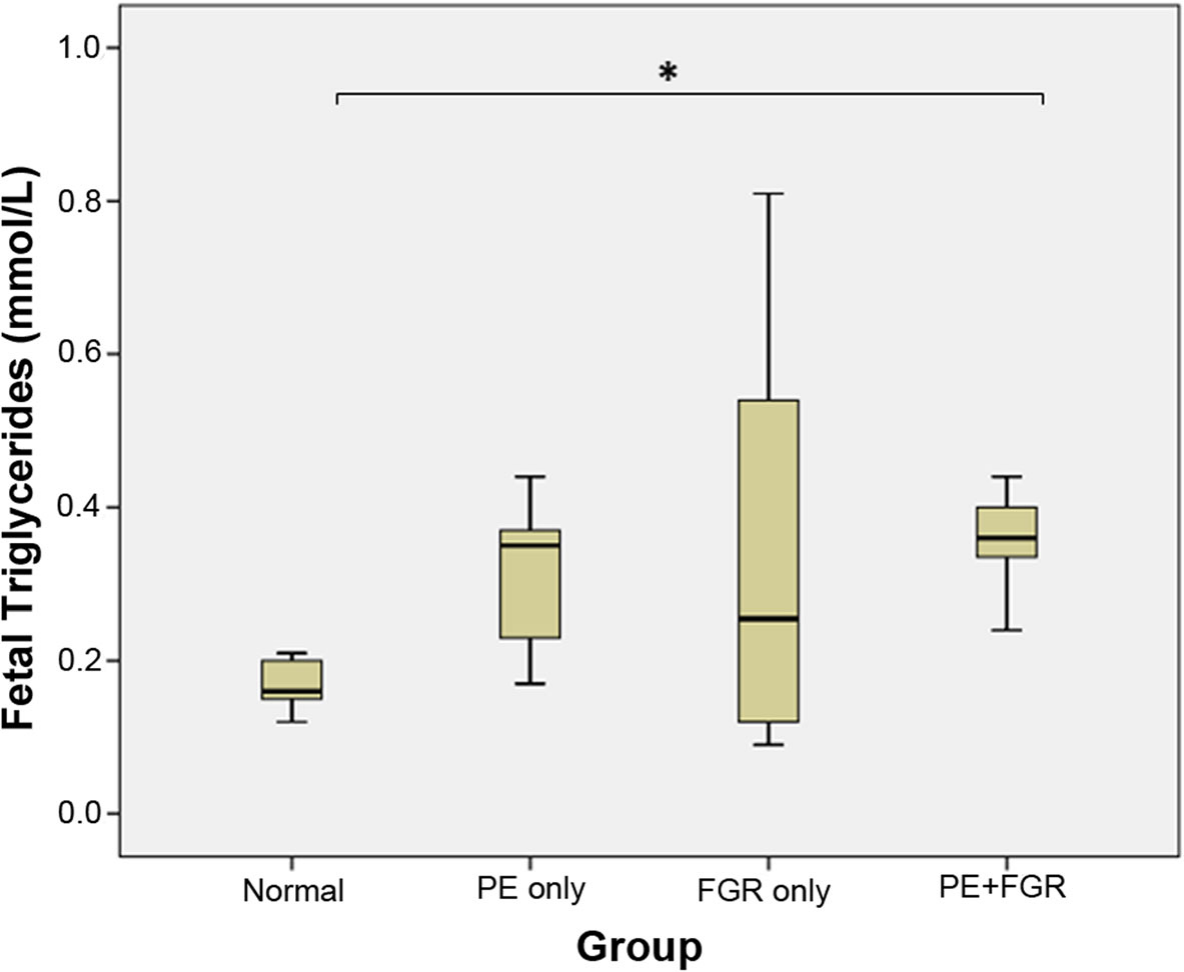
Comparison of fetal/ cord blood total triglyceride (TG) levels between clinical groups

**Fig. 3:**
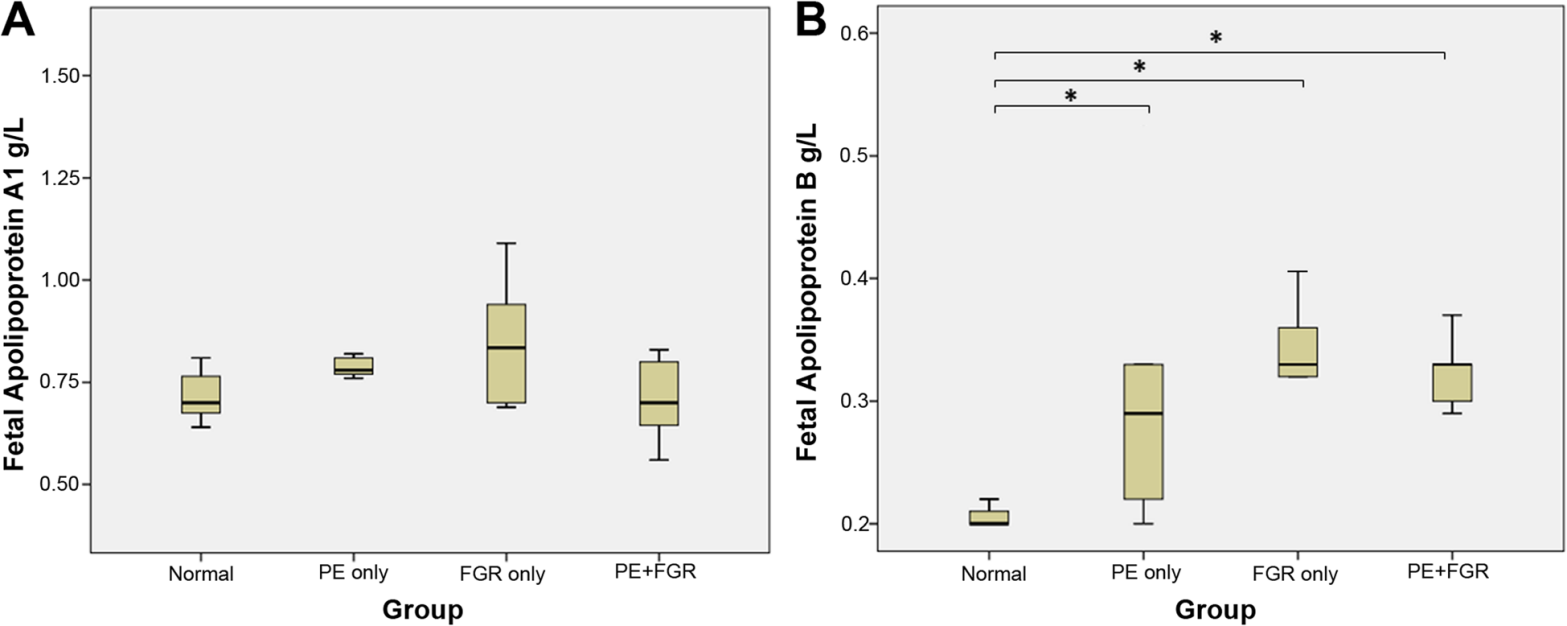
Comparison of fetal/cord blood **a** ApoA1 and **b** ApoB between clinical groups

The fetal lipid profiles of each clinical group are summarized in Table 4 and Figs. 2 and 3. Median fetal TG levels were significantly elevated with the PE + FGR group only (Fig. 2). No differences were noted in the cholesterol levels TC, HDL and LDL between groups. The ApoA1 levels were not different between groups (Fig. 3a). Significantly higher ApoB levels were noted between normal pregnancy and all three pathological groups PE, FGR and PE + FGR, and their levels between the pathological groups were comparable (Fig. 3b). The fetal Apolipoprotein B/A1 ratio (ApoB/ApoA1) was significantly elevated in FGR and PE + FGR (Table 4).

**Table 4:** Fetal lipid profile in normal pregnancy and pregnancies complicated by preeclampsia, intrauterine fetal growth restriction and a combination of PE and FGR

Fetal Data	Group			
	Normal	PE only	FGR only	PE + FGR
	Median (Quartiles 25, 75)	Median (Quartiles 25, 75)	Median (Quartiles 25, 75)	Median (Quartiles 25, 75)
Number of samples	7	5	8	10
TC mmol/L	1.5 (1.3, 2.0)	1.8 (1.5, 2.0)	1.8 (1.5, 2.2)	1.8 (1.5, 2.2)
HDL mmol/L	0.6 (0.4, 0.7)	0.7 (0.7, 0.8)	0.7 (0.5, 1.1)	0.7 (0.4, 0.7)
LDL mmol/L	0.8 (0.6, 1.0)	0.8 (0.7, 1.1)	1.0 (0.8, 1.0)	1.0 (0.9, 1.1)
TC/HDL ratio	2.5 (2.2, 3.2)	2.3 (2.2, 2.9)	2.55 (2.0, 3.4)	3.1 (2.6, 3.5)
TG mmol/L	0.16 (0.14, 0.21)	0.35 (0.23, 0.37)	0.26 (0.12, 0.54)	0.36* (0.32, 0.43)
Apo A1 g/L	0.70 (0.66, 0.81)	0.78 (0.77, 0.81)	0.84 (0.70, 0.94)	0.70 (0.60, 0.82)
Apo B g/L	0.20 (0.20, 0.22)	0.29* (0.22, 0.33)	0.33* (0.32, 0.36)	0.33* (0.29, 0.33)
Apo B: Apo A1	0.29 (0.25, 0.30)	0.35 (0.27, 0.42)	0.37* (0.30, 0.47)	0.42* (0.35, 0.53)

Results are presented as median ± interquartile range for each continuous variable. *Statistically significant difference from normal pregnancy *p* < 0.05

## Discussion

In the present study, we have shown that maternal TG levels in PE are higher than normal pregnancy and FGR. Fetal TG was observed to be elevated in PE + FGR with increased ApoB in all pathological pregnancies PE, FGR and PE + FGR as compared to normal pregnancies. While the TG changes in pregnancy complications have been described before, this is the first description of elevated ApoB levels in cord blood at delivery in PE and FGR. Both the maternal and fetal elevated TG levels and fetal ApoB levels may be of relevance in the pathogenesis of increased cardiovascular disease in later life for mother and neonate.

Our finding of increased maternal TG in preeclampsia is consistent with a number of previous studies documenting elevated lipids in normal pregnancy as well as in preeclampsia [23, 25]. We did not however observe a difference in cholesterol levels as described in previous studies [23]. The maternal data are consistent with previous reports of no difference in ApoA1 and ApoB levels in normal and preeclamptic patients [45].

The mechanism whereby pregnancy induces hyperlipidemia has not been fully elucidated. The elevated maternal triglyceride levels may represent pre-existing hyperlipidemia or risk for hyperlipidemia. Excessive elevation in maternal TG levels may have a role in the pathogenesis of PE and may also identify pregnant women at risk of preeclampsia and long-term cardiovascular risk.

These data are in concordance with previous suggestions that human gestation is associated with an ‘atherogenic’ lipid profile that is further enhanced in preeclampsia and that this profile may be a potential contributor to endothelial cell dysfunction [34]. Although in vitro experiments have shown lipid fraction dependent endothelial activation using preeclamptic plasma [46], a mechanism for serum lipid related endothelial dysfunction has not yet been confirmed.

Triglycerides and free fatty acids have been noted to be already elevated in the first and second trimester in preeclamptic women [33]. A significant correlation between gestational age and lipid parameters could not be demonstrated in our study. Since our study was conducted on samples taken from 26 weeks to 40 weeks, mainly in third trimester, this does not exclude gestation related changes in lipid profile in third trimester compared to first or second trimester. There was no correlation between BMI and TG levels, so a confounding effect would be unlikely. Parity and maternal age did not show any correlation with lipid levels.

Constitutional lipid abnormalities have been suggested as one of the maternal predisposing factors for developing preeclampsia [33]. The study has also documented that FGR only is not significantly associated with elevated maternal TG levels when compared to normal pregnancy, although a difference was seen between PE and FGR. This may be one of the few differences notable in the maternal metabolism between PE and FGR, two conditions that show similar placental pathology [47] and circulating angiogenic milieu [48]. It would be interesting to evaluate whether the maternal response to placental disease is influenced by the TG levels especially whether low maternal TG levels are associated with FGR as opposed to high TG levels associated with PE. The known endothelial effects of TG in maternal circulation supports this hypothesis [46].

Triglyceride levels decrease rapidly in the postpartum period, returning to non-pregnant levels during the puerperium, while LDL levels remain elevated for at least six to seven weeks [49]. Postpartum factors can also influence this return, with lactation associated with an earlier or more complete return [50]. We have shown that TG levels are elevated in PE. It is not known whether the postpartum changes in lipid status is altered in PE or FGR, with possible effect on the long-term cardiovascular risk to the mother.

Ethnic differences have been described in lipid profiles in pregnancy with African/Afro-Caribbean pregnant women having lower serum concentrations of TC, LDL, HDL and TG concentrations compared with Caucasian women [51]. The lipid profile in South Eastern and East Asian background has not been defined. The multicultural nature of the patient population in this study may have affected the presented results.

The significant increase in fetal TG in PE + FGR and the associated trend in PE and FGR is consistent with previous findings suggesting dyslipidemia in fetal growth restriction [37]. The analysis is likely to have been affected by the smaller sample numbers with a wide interquartile range and further studies would be beneficial with larger sample numbers to explore this interesting result that may have implications for fetal origins of adult cardiovascular disease.

These results also indicate that while ApoA1 and ApoB levels were not different between clinical groups in the maternal circulation, significant variation existed in the fetal circulations with elevated ApoB levels in PE, FGR and PE + FGR as compared to normal pregnancies. This is the first documentation of elevated ApoB levels in the fetal cord blood at delivery. The data on fetal ApoB levels confirms the findings of a previous study showing elevated Apo B levels in in-utero cordocentesis samples of growth restricted fetuses [40]. Increased ApoB levels and an elevated ApoB/ApoA1 ratio in young adults have been shown to be predictors of atherogenesis in later life. Fetal growth restriction and low growth in the first years of life has been linked to cardiovascular disease in adulthood [39, 52]. The elevated ApoB levels in the cord blood may play a part in the pathogenesis of the increased long-term cardiovascular risk associated with FGR. Familial defect in apolipoprotein B-100 is associated with moderate to severe hypercholesterolemia [53]. Maternal and fetal gene polymorphisms for lipid metabolism should be investigated for their potential contribution to predisposition to PE and FGR.

The study was limited by small patient numbers and the cross-sectional design. It is possible that the results may be more conclusive with larger patient numbers. The authors acknowledge that the study design does not permit distinction between cause and effect and has been used to generate hypotheses from the observed results to generate hypothesis to be studied via prospective cohort or other studies. The authors have presented gestational age at maternal sample collection and gestational age at delivery separately. In the study design, the cases were controlled by antenatal maternal sample collection of two matched gestational controls for each gestational range to reduce the potential confounding effects of gestation. There is no difference in the maternal sample collection gestational age between normal, PE and FGR groups. There is a difference in gestational age at sample collection between normal and PE + FGR that may have had an effect on the results. The inability to harvest cord blood samples from some recruited pregnancies have reduced the number of fetal samples available for analysis. However, the comparison of the fetal samples was between the available samples from the fetal groups rather than a comparison with the maternal groups and the comparison was considered reasonable.

All study groups show a high caesarean rate. The indications for caesarean delivery in the control group, normal pregnancy were breech presentations and previous caesarean delivery. This group was chosen to reduce the effects of labour on any fetal blood samples match the caesarean rate in the pathological groups. Since the maternal blood was collected prior to delivery, the mode of delivery should not affect the maternal results. The definition of FGR in this study included abnormal umbilical artery Doppler studies. It is not unexpected to see a high caesarean rate in preterm preeclampsia and early delivery for FGR with and without preeclampsia.

The LDL levels were obtained by calculation using the Friedwald equation which assumes that the composition of lipoproteins in pregnancy is the same as in normal metabolic states and also includes intermediate density cholesterol. The calculation is generally applicable to triglyceride levels < 4.5 mmol/L. The TG levels in pregnancy are often significantly higher than this range, leading to the LDL calculation to be less reliable in these patients. The Friedewald equation tends to underestimate LDL-C when triglycerides are elevated as is the case in pregnancy. The LDL levels in this study may therefore be an underestimate.

The findings will have to be tested in pregnancies complicated by pre-existing hypertension and diabetes as well as gestational diabetes to define the applicability of the results to these populations.

Longitudinal studies in pregnancy to evaluate the benefit of such a screening method for preeclampsia in the current pregnancy and the long-term benefits of identifying mothers with pre-existing hyperlipidemia or at risk of long-term hyperlipidemia should be separately evaluated. This approach may parallel screening programs into hyperinsulinemia and gestational diabetes in pregnancy, currently utilized to identify at risk pregnant women for diabetes in pregnancy as well as long term.

Further study should be conducted to evaluate the value of ApoB levels in the cord blood samples of growth restricted fetuses to identify risk of long-term atherosclerosis. Testing of ApoB levels in childhood or adolescent/young adults and correlating with birth weight may give an indication of whether a persistent ApoB level contributes to the pathogenesis of long-term cardiovascular disease.

## Conclusion

In a prospective cross-sectional case control study on maternal and fetal lipid levels in preeclampsia and fetal growth restriction, the authors have demonstrated not only that maternal TG levels are increased in preeclampsia but also that fetal TG and ApoB are elevated in these conditions.

The presented research raises interesting hypotheses regarding the pathogenesis and predisposition to preeclampsia and cardiovascular disease. Elevation in maternal TG levels may have a role in the pathogenesis of PE. The implications of elevated maternal and fetal TG levels and elevated fetal Apolipoprotein B levels deserves further exploration of their role in long term cardiovascular risk in the mother as well as the offspring.

## Data Availability

The data from this study is available on reasonable request from the study authors upon approval of the Western Sydney Local Health District Ethics Committee.

## Abbreviations

ApoA: Apolipoprotein A
ApoB: Apolipoprotein B
CVD: Cardiovascular disease
FGR: Fetal growth restriction
HDL: High density lipoprotein
LDL: Low density lipoprotein
PE: Preeclampsia
SGA: Small for gestational age
TC: Total Cholesterol
TG: Triglycerides

## References

[1] Bellamy L, Casas JP, Hingorani AD, Williams DJ (2007). Pre-eclampsia and risk of cardiovascular disease and cancer in later life: systematic review and meta-analysis. BMJ.

[2] Piepoli MF, Hoes AW, Agewall S, Albus C, Brotons C, Catapano AL (2016). 2016 European guidelines on cardiovascular disease prevention in clinical practice. Revista Espanola De Cardiologia (English ed).

[3] Vikse BE (2013). Pre-eclampsia and the risk of kidney disease. Lancet.

[4] Basit S, Wohlfahrt J, Boyd HA (2018). Pre-eclampsia and risk of dementia later in life: nationwide cohort study. BMJ.

[5] Barker DJ, Bull AR, Osmond C, Simmonds SJ (1990). Fetal and placental size and risk of hypertension in adult life. BMJ.

[6] Townsend R, Khalil A, Premakumar Y, Allotey J, Snell KIE, Chan C (2019). Prediction of pre-eclampsia: review of reviews. Ultrasound Obstet Gynecol.

[7] Rolnik DL, Wright D, Poon LCY, Syngelaki A, O'Gorman N, de Paco MC (2017). ASPRE trial: performance of screening for preterm pre-eclampsia. Ultrasound Obstet Gynecol.

[8] Al-Amin A, Rolnik DL, Black C, White A, Stolarek C, Brennecke S (2018). Accuracy of second trimester prediction of preterm preeclampsia by three different screening algorithms. Aust N Z J Obstet Gynaecol.

[9] Boullart ACI, de Graaf J, Stalenhoef AF (2012). Serum triglycerides and risk of cardiovascular disease. Biochim Biophys Acta.

[10] Gordon T, Castelli WP, Hjortland MC, Kannel WB, Dawber TR (1977). High density lipoprotein as a protective factor against coronary heart disease. The Framingham Study. Am J Med.

[11] Murphy AJ, Woollard KJ (2010). High-density lipoprotein: a potent inhibitor of inflammation. Clin Exp Pharmacol Physiol.

[12] Dashty M, Motazacker MM, Levels J, de Vries M, Mahmoudi M, Peppelenbosch MP (2014). Proteome of human plasma very low-density lipoprotein and low-density lipoprotein exhibits a link with coagulation and lipid metabolism. Thromb Haemost.

[13] Nielsen LB (1996). Transfer of low density lipoprotein into the arterial wall and risk of atherosclerosis. Atherosclerosis..

[14] Kruth HS (2001). Lipoprotein cholesterol and atherosclerosis. Curr Mol Med.

[15] Goldberg IJ, Eckel RH, McPherson R (2011). Triglycerides and heart disease: still a hypothesis?. Arterioscl Throm Vas..

[16] Kannel WB, Dawber TR, Kagan A, Revotskie N, Stokes J (1961). Factors of risk in the development of coronary heart disease--six year follow-up experience. The Framingham study. Ann Intern Med.

[17] Kannel WB, Gordon T, Castelli WP (1981). Role of lipids and lipoprotein fractions in atherogenesis: the Framingham study. Prog Lipid Res.

[18] Wierzbicki AS, Clarke RE, Viljoen A, Mikhailidis DP (2012). Triglycerides: a case for treatment?. Curr Opin Cardiol.

[19] Barter PJ, Ballantyne CM, Carmena R, Castro Cabezas M, Chapman MJ, Couture P (2006). Apo B versus cholesterol in estimating cardiovascular risk and in guiding therapy: report of the thirty-person/ten-country panel. J Intern Med.

[20] Sniderman AD, Furberg CD, Keech A (2003). Roeters van Lennep JE, Frohlich J, Jungner I, et al. Apolipoproteins versus lipids as indices of coronary risk and as targets for statin treatment. Lancet.

[21] Benn M, Nordestgaard BG, Jensen GB, Tybjaerg-Hansen A (2007). Improving prediction of ischemic cardiovascular disease in the general population using apolipoprotein B: the Copenhagen City heart study. Arterioscl Throm Vas.

[22] Contois JH, Warnick GR, Sniderman AD (2011). Reliability of low-density lipoprotein cholesterol, non-high-density lipoprotein cholesterol, and apolipoprotein B measurement. J Clin Lipidol.

[23] Piechota W, Staszewski A (1992). Reference ranges of lipids and apolipoproteins in pregnancy. Eur J Obstet Gynecol Reprod Biol.

[24] Roy AC, Loke DF, Saha N, Viegas OA, Tay JS, Ratnam SS (1994). Interrelationships of serum paraoxonase, serum lipids and apolipoproteins in normal pregnancy. A longitudinal study. Gynecol Obstet Investig.

[25] Rymer J, Constable S, Lumb P, Crook M (2002). Serum lipoprotein (a) and apolipoproteins during pregnancy and postpartum in normal women. J Obstet Gynaecol.

[26] Hillman L, Schonfeld G, Miller JP, Wulff G (1975). Apolipoproteins in human pregnancy. Metabolism.

[27] Montes A, Walden CE, Knopp RH, Cheung M, Chapman MB, Albers JJ (1984). Physiologic and supraphysiologic increases in lipoprotein lipids and apoproteins in late pregnancy and postpartum. Possible markers for the diagnosis of “prelipemia”. Arteriosclerosis.

[28] Wiznitzer A, Mayer A, Novack V, Sheiner E, Gilutz H, Malhotra A (2009). Association of lipid levels during gestation with preeclampsia and gestational diabetes mellitus: a population-based study. Am J Obstet Gynecol.

[29] Hubel CA, McLaughlin MK, Evans RW, Hauth BA, Sims CJ, Roberts JM (1996). Fasting serum triglycerides, free fatty acids, and malondialdehyde are increased in preeclampsia, are positively correlated, and decrease within 48 hours post partum. Am J Obstet Gynecol.

[30] Chalas J, Audibert F, Francoual J, Le Bihan B, Frydman R, Lindenbaum A (2002). Concentrations of apolipoproteins E, C2, and C3 and lipid profile in preeclampsia. Hypertens Pregnancy.

[31] Enquobahrie DA, Williams MA, Butler CL, Frederick IO, Miller RS, Luthy DA (2004). Maternal plasma lipid concentrations in early pregnancy and risk of preeclampsia. Am J Hypertens.

[32] Bayhan G, Kocyigit Y, Atamer A, Atamer Y, Akkus Z (2005). Potential atherogenic roles of lipids, lipoprotein(a) and lipid peroxidation in preeclampsia. Gynecol Endocrinol.

[33] Gratacos E (2000). Lipid-mediated endothelial dysfunction: a common factor to preeclampsia and chronic vascular disease. Eur J Obstet Gynecol Reprod Biol.

[34] Belo L, Caslake M, Gaffney D, Santos-Silva A, Pereira-Leite L, Quintanilha A (2002). Changes in LDL size and HDL concentration in normal and preeclamptic pregnancies. Atherosclerosis.

[35] Ness RB, Sibai BM (2006). Shared and disparate components of the pathophysiologies of fetal growth restriction and preeclampsia. Am J Obstet Gynecol.

[36] Gordijn SJ, Beune IM, Thilaganathan B, Papageorghiou A, Baschat AA, Baker PN (2016). Consensus definition of fetal growth restriction: a Delphi procedure. Ultrasound Obstet Gynecol.

[37] Pecks U, Brieger M, Schiessl B, Bauerschlag DO, Piroth D, Bruno B (2012). Maternal and fetal cord blood lipids in intrauterine growth restriction. J Perinat Med.

[38] Herrera E, Amusquivar E, Lopez-Soldado I, Ortega H (2006). Maternal lipid metabolism and placental lipid transfer. Horm Res.

[39] Barker DJ, Winter PD, Osmond C, Margetts B, Simmonds SJ (1989). Weight in infancy and death from ischaemic heart disease. Lancet.

[40] Radunovic N, Kuczynski E, Rosen T, Dukanac J, Petkovic S, Lockwood CJ (2000). Plasma apolipoprotein A-I and B concentrations in growth-retarded fetuses: a link between low birth weight and adult atherosclerosis. J Clin Endocrinol Metab.

[41] Kim SM, Lee SM, Kim SJ, Kim BJ, Shin S, Kim JR (2017). Cord and maternal sera from small neonates share dysfunctional lipoproteins with proatherogenic properties: Evidence for Barker's hypothesis. J Clin Lipidol.

[42] Tranquilli AL, Dekker G, Magee L, Roberts J, Sibai BM, Steyn W (2014). The classification, diagnosis and management of the hypertensive disorders of pregnancy: a revised statement from the ISSHP. Pregnancy Hypertens.

[43] Martin SS, Blaha MJ, Elshazly MB, Brinton EA, Toth PP, McEvoy JW (2013). Friedewald-estimated versus directly measured low-density lipoprotein cholesterol and treatment implications. J Am Coll Cardiol.

[44] Kolovou GD, Mikhailidis DP, Kovar J, Lairon D, Nordestgaard BG, Ooi TC (2011). Assessment and clinical relevance of non-fasting and postprandial triglycerides: an expert panel statement. Curr Vasc Pharmacol.

[45] Var A, Kuscu NK, Koyuncu F, Uyanik BS, Onur E, Yildirim Y (2003). Atherogenic profile in preeclampsia. Arch Gynecol Obstet.

[46] Davidge ST, Signorella AP, Hubel CA, Lykins DL, Roberts JM (1996). Distinct factors in plasma of preeclamptic women increase endothelial nitric oxide or prostacyclin. Hypertension.

[47] Alahakoon TI, Zhang W, Arbuckle S, Zhang K, Lee V (2018). Reduced angiogenic factor expression in intrauterine fetal growth restriction using semiquantitative immunohistochemistry and digital image analysis. J Obstet Gynaecol Res.

[48] Alahakoon TI, Zhang W, Trudinger BJ, Lee VW (2014). Discordant clinical presentations of preeclampsia and intrauterine fetal growth restriction with similar pro- and anti-angiogenic profiles. J Matern Fetal Neonatal Med.

[49] Potter JM, Nestel PJ (1979). The hyperlipidemia of pregnancy in normal and complicated pregnancies. Am J Obstet Gynecol.

[50] Stuebe AM, Rich-Edwards JW (2009). The reset hypothesis: lactation and maternal metabolism. Am J Perinatol.

[51] Koukkou E, Watts GF, Mazurkiewicz J, Lowy C (1994). Ethnic differences in lipid and lipoprotein metabolism in pregnant women of African and Caucasian origin. J Clin Pathol.

[52] Barker DJ (1990). The fetal and infant origins of adult disease. BMJ.

[53] Innerarity TL, Mahley RW, Weisgraber KH, Bersot TP, Krauss RM, Vega GL (1990). Familial defective apolipoprotein B-100: a mutation of apolipoprotein B that causes hypercholesterolemia. J Lipid Res.

